# Beyond the canvas: a Delphi-driven rubric for unlocking artistic giftedness

**DOI:** 10.3389/fpsyg.2026.1961771

**Published:** 2026-09-23

**Authors:** Yusra Zaki Aboud, Rommel Mahmoud AlAli, Rana Koshha, Mamdouh Mosaad Helali

**Affiliations:** 1 The National Research Center for Giftedness and Creativity, King Faisal University, Al-Ahsa, Saudi Arabia; 2 Evaluation and Assessment Department, Damascus University, Damascus, Syria

**Keywords:** artistic talent, Delphi method, gifted rating scale, rubric, visual arts, criterion-referenced assessment, Saudi Arabia

## Abstract

**Introduction:**

The current study aims to provide a reference and methodological evidence-based tool with particular criteria for identifying artistically gifted students at the intermediate and secondary levels in the Kingdom of Saudi Arabia.

**Methods:**

To achieve the study goals, a modified Delphi design was used, which combined qualitative and quantitative research methodologies to obtain a consensus. The study sample included 120 experts—including art professors, professional visual artists, and art critics-who took part in three rounds. The first round involved thematic analysis of open-ended replies using NVivo 12 software (with an intercoder reliability coefficient of κ = 0.88), resulting in an initial set of criteria. In the second round, experts rated each criterion on a Likert scale and provided qualitative feedback. They then re-evaluated the criteria in the third round using statistical indicators (agreement rates, median, and interquartile range). Written justifications were provided for scores below 4.

**Results:**

The pre-established consensus criterion was set at an agreement rate of ≥85%, defined as picking “agree” or “strongly agree”. The “Gifted Rating Scale for Artistic Talent” (GRS-AT) was created specifically to identify artistic talent in visual arts within Saudi schools; as such, it is the first performance-based assessment tool developed through expert consensus that provides teachers with a structured means for the systematic identification of artistically gifted students—a population that has long been overlooked.

**Discussion:**

Future research is needed to empirically validate the scale through field testing, including an examination of construct validity, criterion validity, inter-rater reliability, and predictive validity.

## 1 Introduction

Educational institutions aspire for holistic development in students, and including the arts into school curricula is a critical component of achieving this goal. A increasing body of study evidence highlights the value of arts education in boosting learners' cognitive, emotional, and motor development (Al-Nasser, 2014; Al-Ghamdi, 2022; Aboud, 2023a; Ji et al., 2026). The arts can help gifted students improve their emotional expression, creative problem-solving skills, insight, vision, and reflective thinking, which are often overlooked in traditional education (Al-Hussein and Aboud, 2024; Drake and Winner, 2021; Brajčić et al., 2018; Barchana-Lorand, 2015). Furthermore, art should not be considered as a topic that fills empty slots in the school schedule; rather, it can be compared to a “silent savior” who takes the hands of artistically inclined pupils when their grades do not meet the equations of success, transforming dissatisfaction into creativity. It is worth mentioning that when school resources are low, funding for arts instruction is frequently curtailed or eliminated (National Coalition for Core Arts Standards, 2014). When grades fail, these kids may find solace in their artistic creations, transforming the classroom from a prison of numbers into a sanctuary where they can recreate themselves, each in their own language that only they understand (Abdulhafeez, 2021).

Despite the established educational usefulness of the arts, particularly for bright children (Hetland and Winner, 2001; Cukierkorn, 2008), their incorporation into the Saudi educational system has generally been limited. Previous educational priorities and cultural conventions contributed to a long lack of formal visual arts training from national curricula, such as drawing and sculpting. This resulted in a deliberate neglect of a field regarded as one of the most important pillars in nurturing creativity and visual culture in Saudi society. However, recent signs of a qualitative transformation to support arts education within educational institutions have emerged, aligning with the Kingdom's Vision 2030 and recognizing the importance of creativity and cultural participation in the development of a knowledge-based economy.

Even with these achievements, visual arts education is still in its early phases; the integration of the arts into school curriculum has not yet reached the needed level of maturity, nor have appropriate resources been assigned in proportion to its value. Given the relative novelty of interest in the arts in Saudi schools in general, there is a scarcity of Saudi studies concerned with assessing and identifying artistically gifted students using rigorous scientific instruments; the available tools in this field do not go beyond simple methods based on nomination (Al-Nasser, 2014) and self-nomination (Abu Nasser et al., 2019). In contrast, Abdulhafeez's (2021) study aimed to identify the elements that contributed to the refining of creative talents among a select group of international visual artists, with the goal of using these factors to develop a vision for developing artistically gifted kids.

The current study addresses this important research gap by developing a system for identifying and evaluating artistically gifted students in Saudi schools. This is accomplished through the development of the ‘Gifted Rating Scale-Art' (GRS-AT), which is proposed as a tool for detecting artistically gifted individuals pending further empirical validation in Saudi Arabia pending further empirical validation through field testing.

## 2 Study problem

Education in most educational systems focuses on the attention to discovering and nurturing the gifted in academic sciences only, neglecting a highly significant aspect, which is paying attention to the creatively and artistically gifted. They do not receive their fair share of attention and care as is the case in other academic fields. Despite the indications of scientists and researchers regarding the importance of art, as advocated by Read, 1996, as cited in Barchana-Lorand (2015) in his statement “on the importance of art as a ground for human knowledge,” he emphasized that art is considered a common denominator among the various facets of human knowledge (In: Barchana-Lorand, 2015). Every knowledge and experience has its aesthetic aspect, which cannot be overlooked; rather, this aesthetic aspect is closer to human nature than the dry aspects contained in that knowledge. Therefore, all education can be conducted through the aesthetic entrance. He formulated this in his famous book “Education through Art” (Barchana-Lorand, 2015).

On the other hand, despite the establishment of an institution concerned with the care of gifted students in the Kingdom of Saudi Arabia, namely the King Abdulaziz and His Companions Foundation for Giftedness and Creativity (Mawhiba), and its provision of distinguished care programs such as mentorship programs, excellence programs, and local and international enrichment programs for gifted students, as well as the creation of special classes for the gifted, the foundation's focus has been solely on scientific fields such as science and mathematics, excluding the field of art education and fine arts. The situation is no different for the gifted care centers established for the same purpose by the Ministry of Education in Saudi Arabia, according to statements from two officials in the Al-Ahsa Education Department during a personal interview conducted with them. They both agreed on the lack of familiarity among art education teachers on how to deal with artistically gifted students; this is a result of the absence of a mechanism or a vision for methods to refine their artistic skills. Furthermore, the efforts made to nurture artistically gifted students, whether in those centers or even in schools, are few and limited on one hand, and vary among art education teachers according to their degree of interest in them on the other. They also mentioned that even when teachers are interested in nurturing artistically gifted students, this care does not go beyond allowing them to participate in some limited art competitions held occasionally during the school year, and using words of praise and commendation for them in front of their peers in the classroom. They also agreed that the absence of a clear vision or strategy for nurturing artistically gifted students is the main problem that has created teachers who are unqualified to nurture artistically gifted students. Furthermore, Saudi student care centers lack a specific mechanism for the artistically gifted student (Abdulhafeez, 2021).

## 3 Literature

### 3.1 Developing the talents of artistically gifted learners

Developing the talents of artistically gifted learners requires a distinct approach that acknowledges the unique characteristics and needs of this population. Unlike academically gifted students, who may benefit from acceleration and curriculum compacting in traditional subjects, artistically gifted students often require more time for creative exploration and project completion (Abdulhafeez, 2021). Research on talent development in the visual arts emphasizes the importance of providing differentiated instruction that balances enrichment (exposure to diverse artistic media and techniques) with acceleration (targeted development of specific talent areas) (Drake and Winner, 2021). Art teachers play a critical role in this process through the creation of studio-based learning environments that incorporate student choice, individualized feedback, and exposure to advanced artistic materials (Ji et al., 2026; Groenendijk et al., 2020).

Furthermore, general classroom teachers can support artistically gifted students by integrating activities that engage their artistic strengths into academic instruction, thereby benefiting all learners (Haroutounian, 2017). This professional learning should be embedded within broader gifted education training to ensure that all teachers are equipped to recognize and nurture artistic talent. Although most art teachers have training and a background in artistic fields, they often lack professional training specifically related to the development of artistically gifted learners' talents (Abdulhafeez, 2021).

While research has focused extensively on academic giftedness, studies examining the effectiveness of differentiation or acceleration options for artistically gifted students remain scarce. In traditional subjects such as mathematics, a gifted student may require less time to complete a task; however, the gifted artist may need more time to complete a project in art class. Therefore, research findings in other disciplines may not apply directly to the arts (Abdulhafeez, 2021). Generally, especially at early ages, a combination of enrichment (exploring a variety of artistic media) and acceleration (developing specific talent areas) is justified. As students' progress in developing their talents, the focus tends to shift more toward their specific talent area and specialization within that artistic field (Drake and Winner, 2021).

Art teachers can provide differentiated instruction and respond to the needs of all learners by working to create a studio model in the art classroom (Groenendijk et al., 2020). This approach allows them to incorporate more student choices and differentiate content and product. Such practices would benefit talented artists in their classrooms by providing opportunities for expressing their talents and exposure to advanced artistic materials (Ji et al., 2026). General classroom teachers can also benefit from specific professional learning regarding the development of talented gifted students. Although they may not be responsible for the direct development of artistic talent within their Arabic, mathematics, or science classrooms, they may be able to use the strengths of these gifted students in their teaching. By incorporating activities that engage the strengths of artistically gifted students, they can better teach all students (Haroutounian, 2017). This professional learning can be integrated into general professional learning about gifted education for teachers, specifically referring to learners with talents in the arts and the strategies that will benefit them.

### 3.2 Artistically gifted students and their characteristics

Studies confirm the existence of a wide diversity of innate abilities in any field, and that children with high abilities in a particular field share three traits: speed of learning (early maturity), a great passion for learning (a strong desire for mastery), and the difference of their learning methods from ordinary children (excellence in their style). In the visual arts, high abilities are most often observed in the ability to draw realistically (National Assembly of State Art Associations, 2006). We show that this ability is independent of IQ, and we provide some evidence of its association with local processing skills. Therefore, it is possible that gifted child artists take two paths: one is the path of early representation/realism, and the other is the path of early abstraction. We believe that the ability to depict the world realistically should be considered only one among many indicators of talent in the visual arts. Some talented young artists focus on the realistic depiction of objects and are influenced by Western spatial conventions. Others may focus on using art to creatively depict visual narratives, through the use of themes and variations, humor, paradoxes, puns, metaphors, and depth (Clark and Zimmerman, 2004).

Artistically gifted students possess well-developed drawing skills, high cognitive abilities, interest, and motivation. They also possess other characteristics related to intensity of focus, early mastery of cultural forms typical of artistic excellence, production of a large volume of work over a sustained period, and thematically specialized work (Stalker, 1981). These students also create imaginary worlds, engage intensively in a specific field, and experiment with spatial and naturalistic depiction (Stalker, 1981). Clark (1993) found that artistically gifted children began representational drawing 1 to 2 years earlier than their peers, showed a high degree of creativity and originality, demonstrated deep commitment, and drew in qualitatively different ways from their peers. In research on world-class artists, Zidan and Farag (2024) found that, as young children, these artists did not show exceptional abilities. However, they did show inventiveness and were able to depict subjects accurately and with enthusiasm (Clark and Zimmerman, 2004).

Milbrath (as cited in Cukierkorn, 2008) argues that artistically gifted children perceive the world differently from their average peers, who depict things as they see and understand them without representing the actual visual properties of the world around them. In contrast, gifted children depict the world realistically and have the ability to encode visual information with greater precision. Clark and Zimmerman (2004), on the other hand, believe that superior drawing abilities and painstaking attention to detail are necessary traits of artistically inclined people. They advance their talent faster than their ordinary counterparts. Ji et al. (2026) contend that artistically gifted individuals are distinguished by their rapid learning of artistic talents and techniques. They can focus on a single artistic field and broaden their knowledge based on their own interests. They also have excellent recall, can grasp basic artistic ideas, and comprehend complex phenomena. Furthermore, they possess a high level of metacognitive knowledge and skills (Brajčić et al., 2018).

Hetland and Winner (2001) argue that the potential of artistically gifted individuals lies not only in their exceptional ability to express themselves artistically, but also in their capacity to achieve realism in their drawings. This realism is manifested in their ability to observe and remember visual details, complete structures, and convey three-dimensionality. Their works also demonstrate exceptional non-representational abilities in design, composition, and coloring.

According to Abdulhafeez, artistically gifted individuals possess a highly developed spatial ability, linked to their capacity for visual discrimination between details, shadows, and images; visual memory; and the ability to imagine images in minute detail, recalling them intellectually and expressively. They also possess precise hand-eye coordination (2021). Artistically gifted individuals develop these skills through experience, practice, and observing things in unfamiliar ways, ultimately culminating in a sense of harmony (Al-Shammari and Khalaf, 2018). Barchana-Lorand (2015) argues that artistically gifted individuals are distinguished by their diverse subject matter choices, richer artistic vocabulary compared to their peers, expansive imagination, and exceptional ability to depict movement in their paintings. They consciously combine objects and people, possess meticulous attention to color and contrast, and continuously explore new materials for artistic expression. Furthermore, they have the ability to broaden their interests to include new topics and themes, and they are more adept than their average peers at effectively combining visual observation and memory. They also enjoy solitary artistic activities, are more sensitive to observing unfamiliar subjects and forms, and possess an unconventional artistic approach (Jarosewich et al., 2002).

Gardner (1989) believes that the artistic feeling that is achieved through work and learning, considering that artistic talent cannot be fully understood unless we take into account the factor of “feeling,” which embodies the ability to see beyond the appearance of things, generates a sense of harmony, and stimulates the ability to make aesthetic assessments. Expert assessment helps determine whether observed indicators actually point to exceptional talent, and also helps determine whether the artistically gifted individual can benefit from an intensive systematic development program (Cudina-Obradović, 1991).

### 3.3 Identifying artistically gifted students

The endeavor of identifying students endowed with artistic talent necessitates the discernment of consistent and fundamental behavioral patterns and manifestations that diverge from those exhibited by their typical peers. The predominant methodologies employed for the identification of gifted students encompass teacher observations (Al-Ghamdi, 2022), checklists (Abdulhafeez, 2021), art evaluations (Aboud, 2026), and the insights and evaluations proffered by renowned artists (Brandon, 2000). Checklists frequently incorporate essential attributes characteristic of artistically gifted individuals articulated as declarative statements. For instance, examples of statements extracted from the checklist utilized for the identification of gifted students in the GRS-S scale (formulated by Jarosewich et al., 2002) include: ‘Produces imaginative and/or original art' and ‘Produces imaginative and/or original art.'

Notwithstanding the pivotal function of educators in the identification of artistically gifted youths, initial indicators of their exceptional talent are frequently disregarded owing to educators' constrained capacity to systematically evaluate these distinctive abilities at an early stage, as emphasized by (Groenendijk et al. 2020). In contrast, Clark's Drawing Abilities Test (CDAT) requires a practical demonstration, wherein students engage in the completion of four drawing tasks (Clark and Wilson, 1991). Moreover, the CDAT distinguishes itself from our own scale, the Gifted Rating Scale for Artistically Talented (GRS-AT), with respect to the type of assessment: direct (emphasizing the final product, with sample inquiries such as ‘Draw a house from a distance') vs. indirect (centering on the process), which evaluates the artistic process and behavioral patterns such as ‘Produces imaginative and/or original art' or “Learns artistic techniques quickly.”

## 4 Theoretical framework

At a time when the process of evaluating the gifted was based on the principles of behavioral theory, which focused mainly on individual and collective intelligence tests, achievement tests, lists of behavioral characteristics, and creativity tests, the trend has recently begun to benefit from what the cognitive theory has produced, as the process of evaluating the gifted began. It is directed toward reality and the environment that embraces the process of innovation and creativity and focuses on the processes through which a product or work is created, more than its focus on evaluating the factors of talent and creativity (originality, flexibility, fluency...), and this necessarily necessitated focusing on the competencies of gifted students and monitoring the growth Improving their performance, and involving them in the evaluation process, by developing their ability to practice self-assessment.

Therefore, the evaluation process has become more comprehensive and diverse, and several parties participate in it, starting with the gifted students themselves, to their parents, their peers, in addition to their teachers. The purposes of evaluation have varied in accordance with the new trend, from identifying the strengths and weaknesses in the performance of gifted people, to evaluating the curricula offered to them, in addition to evaluating the programs they enroll in to develop their talent.

In response to these modern trends, the concept of alternative assessment has begun to find a prominent place in the process of assessing talent and creativity, as it is, as we mentioned previously, a fundamental shift in the traditional practices that were prevalent for a long time in measuring and evaluating the gifted toward using more diverse strategies relevant to the life of the gifted student, such as performance assessment., product evaluation, and completion file evaluation (Cukierkorn, 2008; Aboud, 2023b).

Wiggins (1993) is considered the pioneer of the alternative assessment movement in 1998 (Aboud, 2022). Given the novelty of the concept of alternative assessment of giftedness, there have been many terms that refer to it. Referring to the literature on measuring and evaluating the gifted, we notice many terms or concepts that are synonymous with this concept, including realistic or real evaluation, evaluation based on real performance, formative evaluation, curriculum-based evaluation, and qualitative evaluation, but the most common and used name is alternative evaluation (Abu Nasser et al., 2019; Allam, 2000).

There is an issue related to identifying and assessing gifted students using alternative assessment methods, and it relates to talent associated with a field such as talents related to the arts, which are difficult to assess with aptitude, intelligence or even achievement tests, which requires those in charge of gifted programs to collect the work of gifted students in particular, who cannot be assessed with traditional tests, and assess it through more creative and flexible tools such as rubrics or assessment scales (Jarosewich et al., 2002).

Beyond the frameworks of alternative and performance-based assessment, this study is also informed by labeling theory, which provides a lens for understanding the social and psychological implications of identifying students as artistically talented. Research on the specific labeling of students who are highly talented in the arts is limited, and findings from studies on academically gifted students do not necessarily apply to those talented in the arts. The label 'artistically talented' may affect students in a completely different way than the label 'academically gifted,' as artistic talent is often less valued in educational systems that prioritize academic achievement (Kay, 2008). Consequently, many researchers are investigating conceptions of giftedness and talent among students participating in artistic and academic programs, including their recollections and interpretations of previous labeling experiences and their perceptions of others' reactions to being labeled (Kay, 2008).

Although theory is not well developed in what might be called the social psychology of gifted and talented children, a parallel body of theoretical formulations has evolved in the social psychology of students with disabilities (Kay, 2008). One of the most widely used frameworks is labeling theory. Originally developed by Becker (1963) and subsequently applied in educational contexts, labeling theory suggests that individuals who are labeled as ‘different' may experience altered expectations and treatment from others, which in turn shapes their self-concept and behavior. When applied to gifted and talented students, labeling theory suggests that others are likely to react differently to persons labeled ‘gifted'; labeling effects may result in patterns of behavior and experiences that can lead to life trajectories very different from those who are not labeled (Kay, 2008).

In the context of this study, labeling theory provides a useful framework for examining how artistically talented individuals are identified, and how the identification process itself may influence their artistic development and self-perception. This theoretical lens complements the alternative assessment approach by highlighting the importance of thoughtful, accurate, and developmentally appropriate identification practices that avoid the potential negative consequences of labeling while maximizing the benefits of recognition and support.

## 5 Method

### 5.1 Methodology

#### 5.1.1 Overall design: a modified Delphi study with integrated qualitative and quantitative stages

This study employed a modified version of the Delphi method to develop a consensus-based, criterion-referenced assessment tool for identifying artistically gifted students at the intermediate and secondary school levels in Saudi Arabia. The Delphi method is a structured, iterative group communication technique designed to reach expert consensus on complex issues through multiple rounds that involve submitting anonymous opinions, presenting controlled statistical summaries, and allowing for refinements in each round. Unlike focus groups or discussion groups, the Delphi method mitigates the influence of dominant individuals, limits inappropriate or biased communication, and alleviates group pressure to reach a consensus (Chu and Ke, 2017).

The Delphi method is a consensus-building approach that relies on iterative rounds to transform individual expert opinions into a collective agreement. Consequently, it is not formally classified as a sequential mixed-methods design in the strict sense of the term (such as sequential exploratory or explanatory designs), even though it inherently incorporates—through its successive rounds—both qualitative elements (such as open-ended exploration and thematic analysis) and quantitative elements (such as Likert-scale ratings). The use of the “Delphi method” in this study is supported by several justifications.

First, experts can express their thoughts without knowing each other's identities, ensuring privacy and reducing the impact of peer pressure. Second, the system allows for structured feedback after each round, which is supported by statistical data like levels of agreement, medians, and interquartile ranges, as well as anonymous written comments. This allows specialists to reconsider their opinions and judgments in light of the group's perspectives, resulting in the formation of uniform and credible standards by the end of the three rounds.

### 5.2 Delphi process

Using the Delphi technique, the researchers sought to establish, quantify, and validate the criteria for a talent assessment scale. This procedure was divided into three phases, with each round building on the results of the previous one to improve outcomes. It involved 120 specialists, including art professors, visual artists, and art critics. In the first phase, which aimed to develop qualitative standards, the experts supplied written descriptions of ideal performance criteria for the artistically gifted student, which the research team examined and objectively analyzed. The first round produced a preliminary list of 21 critical criteria spread across six groups. In the second round, which aimed at quantitative evaluation and qualitative improvement, the experts evaluated each of the previous preliminary criteria using a five-point Likert scale (1 = strongly disagree to 5 = strongly agree), and they proposed improvement modifications or new criteria, supported by the calculation of descriptive statistics for each criterion, including agreement rate, median, and interquartile range. The third round focused on final verification and qualitative justification, with the aggregated results—including statistical summaries and anonymized opinions and comments from the second phase—presented to the participating experts. They were asked to re-evaluate each criterion on a five-point Likert scale, and to submit written justifications for any criterion rated less than four points. The researchers set a consensus threshold of 85%, which meant that 85% or more of the experts had to select “Agree” or “Strongly agree.” As a result, only the criteria that met the 85% requirement by the end of the third round were kept, yielding six criteria—each evaluated on a four-level performance scale: poor, emerging, competent, or excellent (Chu, 2024). Thus, the final measurement tool (the proposed evaluation scale) was created by combining quantitative consensus (approval of 85% or more) with qualitative insights (such as objective descriptions and expert comments).

#### 5.2.1 Qualitative data collection and analysis in rounds 1 and 2

To collect data in the first round, semi-structured interviews (conducted via Zoom and voice messaging via WhatsApp) were utilized in addition to open-ended questionnaires, allowing both researchers and experts to debate questions in depth and explain any points of uncertainty. The experts were tasked with identifying the key characteristics of visually gifted pupils and describing the ideal performance requirements for each area of artistic talent. All qualitative data was transcribed and analyzed using NVivo 12 software and thematic analysis approach (Braun and Clarke, 2006). The first coding process was carried out by two separate coders, who discussed inconsistencies in order to obtain consensus. A third coder then coded a random sample of the data to ensure coding reliability, resulting in a Cohen's kappa coefficient of 0.88, indicating a high level of inter-rater reliability (Cohen, 1960). The thematic analysis identified six main themes—authenticity and creativity; technical competence and craftsmanship; composition and design; developmental significance; critical thinking and self-reflection; and commitment and motivation—as well as 21 sub-themes, which served as the foundation for the second round's draft standards.

#### 5.2.2 Quantitative data collection and analysis (Rounds 2 and 3)

Each expert was given a questionnaire comprising the 21 preliminary criteria from the second round and asked to score their level of agreement on a five-point Likert scale (1 = strongly disagree, 2 = disagree, 3 = neutral, 4 = agree, and 5 = highly agree). Descriptive data were calculated for each criterion, including the agreement rate (the proportion of participants who chose a score of 4 or 5), median, and interquartile range (IQR). The study team gathered and analyzed anonymous written feedback and suggestions. The third round entailed giving the experts with a summary report for each criterion that included the amount of agreement recorded in the second round, the median and interquartile range, as well as reviewed comments on the criteria's representativeness. Based on this report, the experts re-evaluated each criterion on the same five-point Likert scale, providing written reasoning for any rating of 3 or lower. Following the third round, agreement levels were recalculated. All 21 criteria met the pre-established consensus standard (agreement level ≥ 85%); hence no criteria were eliminated.

#### 5.2.3 Integration of qualitative and quantitative data

The study did not follow a systematic design based on “mixed methods” that combine quantitative and qualitative approaches, but integration of these two components was nevertheless accomplished over two phases: first, the transition from the qualitative phase to the quantitative phase, where the themes found during the first qualitative analysis were transformed into preliminary criteria that were then subjected to quantitative evaluation; and second, the transition from the quantitative phase to the qualitative phase, where the statistical summaries and anonymized comments from the second phase were used as feedback that allowed experts to re-evaluate the criteria in the third phase, with their written justifications being qualitatively analyzed to identify areas of divergence.

The study design is shown in Figure 1. Thus, the final measurement tool (the proposed evaluation scale) was created by combining quantitative consensus (approval of 85% or more) with qualitative insights (such as objective descriptions and expert comments. The 21 sub-criteria across the 6 main dimensions were formulated through a systematic, multi-stage process that combined qualitative and quantitative data from Delphi rounds. Figure 1 below illustrates the formulation process in general terms.

**Figure 1:**
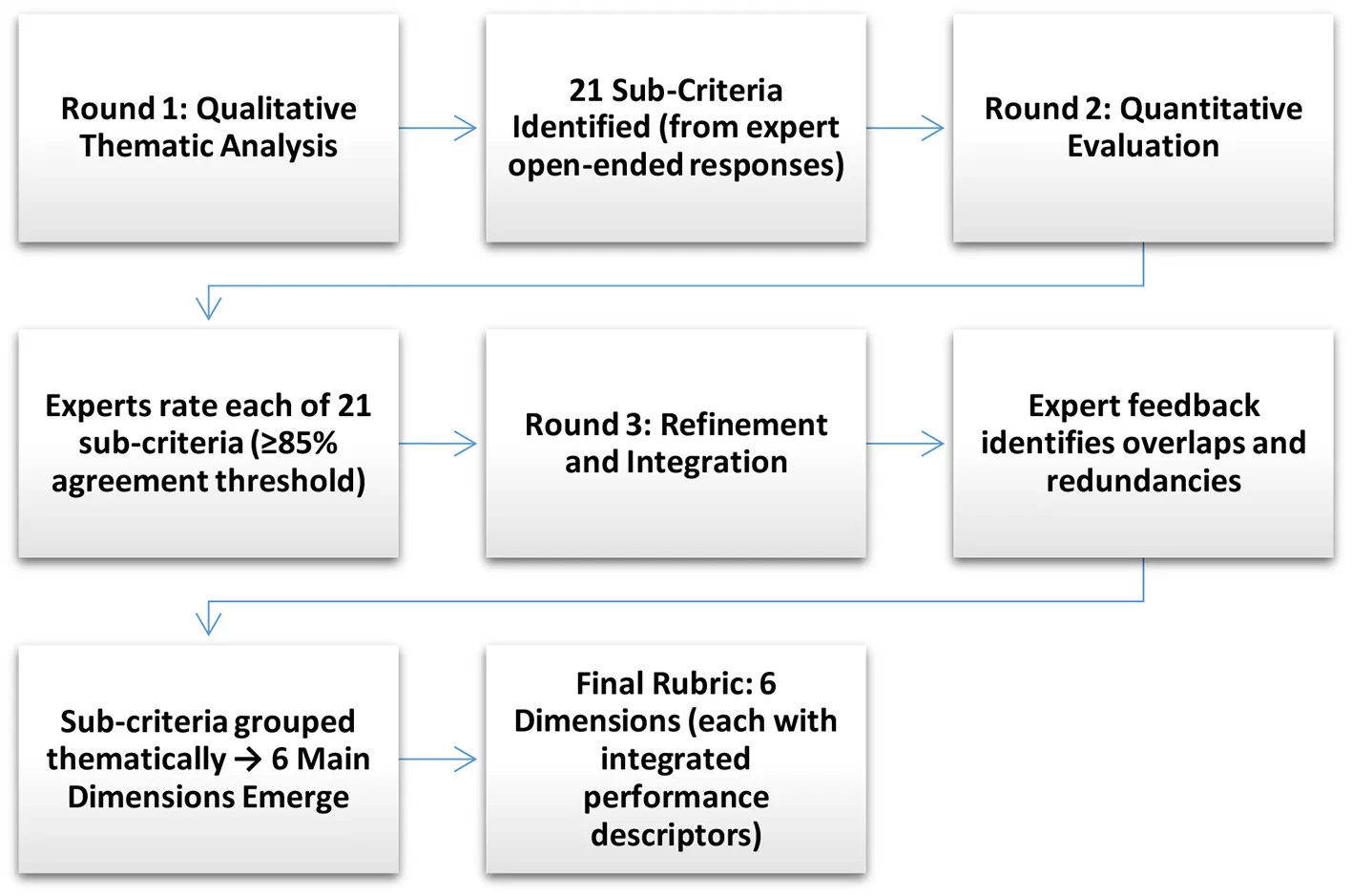
Rubric development process.

### 5.3 Sampling strategy and rationale

#### 5.3.1 Target population and sampling frame

The target population for this Delphi study consisted of experts in the fields of visual arts and art education within the Kingdom of Saudi Arabia. The sampling frame was defined to include three categories of experts: a) Academic experts—professors and faculty members specializing in art education at Saudi universities; b) Professional visual artists—practicing artists with established careers; and c) Art critics—individuals with recognized expertise in art criticism and evaluation. Compared to a conventional Delphi study panel, the sample size of 120 experts is far larger. Although Delphi studies typically use panels with 10 to 50 participants, our bigger sample was purposefully chosen to guaranty participation from all Saudi Arabian regions and enough professional diversity—covering the three expert categories—to allow for insightful subgroup analysis. Additionally, it delivers reliable estimates of central tendency and dispersion for each criterion and takes into account expected attrition over several rounds, guaranteeing a sufficient number of experts remain for subsequent rounds.

In accordance with Delphi literature, the concept of an “expert” was operationalized based on the four criteria proposed by Ismail and Taliep (2023): knowledge and experience regarding the issues under investigation; the ability and willingness to participate; sufficient time for participation; and effective communication skills. Each potential participant was assessed against these criteria prior to inclusion.

#### 5.3.2 Recruitment procedures

Potential participants were identified through multiple channels: institutional databases of art education faculty members from Saudi universities, professional networks, and membership lists of Saudi art associations and cultural organizations.

The recruitment process followed a structured protocol. First, potential participants were contacted via official email with a formal invitation letter explaining the study's purpose, describing the Delphi method and the expected time commitment, confirming confidentiality, and providing a link to the first-round questionnaire. Second, interested individuals completed a brief questionnaire to verify that they met the inclusion criteria. Third, eligible participants provided written informed consent prior to participation.

#### 5.3.3 Sample composition

A total of 186 potential participants were contacted. Of these, 135 expressed interest and were screened for eligibility. A total of 120 met all inclusion criteria and provided informed consent, resulting in an initial panel of 120 experts. Table 1 presents the sample structure regarding professional role and practical experience.

**Table 1:** Sample structure regarding independent variables.

Independent variable	Categories	*n*	%
Professional role	Academics	43	35.8
	Professional artists	55	45.8
	Art critics	22	18.3
Work experience	Up to 5 years	24	20
	6 to 11 years	15	12.5
	12 to 17 years	13	10.8
	18 to 23 years	24	20
	24 to 29 years	21	17.5
	More than 30 years	47	39.2
Total		120	100

#### 5.3.4 Attrition across rounds

Attrition is a well-documented concern in Delphi studies, as the iterative nature of the method requires sustained participant engagement across multiple rounds. We carefully monitored response rates and employed strategies to minimize dropout. Table 2 Displayed response and retention rates across the three Delphi rounds.

**Table 2:** Response and retention rates across the three delphi rounds.

Round	Panel members contacted	Completed responses	Response rate	Cumulative retention
Round 1	120	120	100%	100%
Round 2	120	111	92.5%	92.5%
Round 3	111	107	96.4%	89.2%

As indicated in Table 2, In Round 1, all 120 participants filled out the questionnaire (100% response rate) demonstrating the rigorous screening process and the experts' adherence to the study's goals. In Round 2, nine participants (7.5%) did not complete the questionnaire, citing time limits (*n* = 5), personal reasons (*n* = 3), and a lack of interest (*n* = 1). In Round 3, four more participants (3.6% of Round 2 respondents) did not finish the questionnaire. The overall retention rate from Round 1 to Round 3 was 89.2%, which is higher than the acceptable level for Delphi research is comparable to other multi-round Delphi studies in Saudi educational situations.

To reduce attrition, the research team used several strategies, including clear communication about the three-round structure and time commitment, regular email reminders, flexible response windows, summary feedback after each round to keep participants engaged, and multiple communication channels (Zoom interviews, WhatsApp voice messages, or online questionnaires).

To determine if attrition created systematic bias, we compared the demographic features of completers (*n* = 107) to non-completers (*n* = 13). There were no significant differences in professional role distribution (academics: 36.4% vs. 30.8%; professional artists: 45.8% vs. 46.2%; art critics: 17.8% vs. 23.1%), or regional distribution. The mean years of experience were somewhat higher among completers (18.7 years) than non-completers (14.2 years), but this little difference is unlikely to have influenced the overall consensus findings.

## 6 Result

In alignment with the Delphi methodology, the findings are delineated into three segments: (1) the quantitative consensus produced from the three Delphi rounds, (2) the qualitative themes collected from the experts' remarks, and (3) the final criterion for recognizing artistically gifted pupils.

### 6.1 Quantitative fit: Delphi rounds 1–3

#### 6.1.1 Round 1: generating criteria

In the first round, experts offered open-ended replies to determine criteria for optimal performance among artistically gifted kids. A thematic analysis of these responses yielded an initial set of 21 proposed criteria, divided into six domains: 1) originality and creativity, 2) technical competence and craftsmanship, 3) composition and design, 4) developmental appropriateness, 5) critical thinking and self-reflection, and 6) commitment and motivation.

#### 6.1.2 Round 2: initial quantitative evaluation

In the second round, each expert rated the 21 original criteria on a five-point Likert scale (1 = strongly disagree, 2 = disagree, 3 = neutral, 4 = agree, and 5 = strongly agree). Table 1 shows the percentage of experts that chose “Agree” (4) or “Strongly Agree” (5) for each criterion, along with the median and interquartile range (IQR). Table 3 Display expert agreement on draft criteria (Round 2).

**Table 3:** Expert agreement on draft criteria (round 2).

Criterion no.	Criterion summary	% Agree (4+5)	Median	IQR	Status
1	A wider and more diverse range of subjects	96.2	5	0.5	Original
2	Compositional awareness, rich artistic language	95.8	5	1.0	Original
3	Flowing ideas, expansive imagination	95.3	5	0.5	Original
4	Demonstrates movement and form	96.5	5	0.5	Original
5	Leads teamwork	97.2	5	0.5	Original
6	Precise use of color and contrast	98.1	5	0.5	Original
7	Assembles elements intelligently and consciously	93.7	5	1.0	Original
8	High technical skill	91.7	5	1.0	Original
9	Takes notes through drawing	82.1	4	1.5	Revised in Round 3
10	Excellent visual memory and observation	81.8	4	1.5	Revised in Round 3
11	Interaction, memory, and observation	86.1	4	1.0	Original
12	Motivated and committed to the task for extended periods	93.2	5	1.0	Original
13	Self-critical and strives for perfection	96.8	5	0.5	Original
14	Strong interest in visual art	96.3	5	0.5	Original
15	Interest in figurative and abstract forms. Intuitive awareness of composition. Fluid movement between art mediums.	93.3	5	1.0	Original
16	Enjoys solving complex art tasks	97.1	5	0.5	Original
17	Commits to completing art tasks. Overcomes difficulties in the art process.	94.2	5	1.0	Original
18	Loves to understand and learn from things	90.1	5	1.0	Original
19	A wider and more diverse range of subjects (Duplicate of Criterion 1)	83.2	4	1.5	Merged with Criterion 1 in Round 3
20	Compositional awareness, rich artistic language (Duplicate of Criterion 2)	82.1	4	1.5	Revised in Round 3
21	Flowing ideas, expansive imagination (Duplicate of Criterion 3)	86.1	4	1.0	Merged with Crite

IQR, Interquartile Range.

Standards 19, 20, and 21 were initially formulated to mirror Standards 1, 2, and 3, respectively, in order to capture additional indicators within the same thematic areas. However, during the second round, experts identified these as duplicates and recommended revising or merging them. Specifically, Standard 19 was merged with Standard 1; Standard 20 was revised to incorporate more precise wording regarding 'rich technical language'; and Standard 21 was merged with Standard 3. The final, revised standards are presented in Table 4.

As demonstrated in Table 3, 17 out of the 21 criteria met the pre-established consensus requirement of 85%. Four criterion (9, 10, 19, and 20) had agreement ratings less than 85% in the second round. The experts submitted written comments that included adjustments or merging of specific criteria; as a result of the feedback from the second round, these four criteria were amended and re-evaluated in the third round, and they all met the required consensus threshold (85%). For example, criterion 9—originally defined as ‘taking notes through drawing'—was amended to improve clarity and behavioral precision, resulting in an increase in the agreement rate from 82.1% to 85.0%, allowing it to be preserved on the final scale. Although the exact adjustments to the wording of criteria 10, 19, and 20 were not included in the research paper, they were amended in response to expert suggestions and eventually achieved the required consensus level during the third round.

#### 6.1.3 Round three: final verification and reassessment

Although the document did not disclose the specific wording revisions for criterion 10, 19, and 20, they were also updated based on expert suggestions. The experts examined the second round's results summary (percentages, median, interquartile range, and anonymous comments) and reassessed all 21 criteria. After the third round, all 21 criteria had a consensus level of 85% or higher; the highest consensus rate (97.5%) was recorded for both Criterion 5 (“Leads teamwork”) and Criterion 13 (“Demonstrates self-criticism and a tendency to strive for perfection”), while the lowest consensus rate—though still acceptable—was observed for Criterion 9 (“Takes notes through drawing”) at 85.0% following its revision that passed the consensus threshold in the third round. Criterion No. 18—“Artistically gifted children enjoy solving challenging artistic tasks”—had the highest rating in the “Strongly Agree” category (Score 5); 62.5% of respondents chose “Strongly Agree,” with a 90% total agreement rate. This shows that experts regard a desire for challenge as a critical distinguishing characteristic. Notably, no criteria were left out, and the final set of 21 criteria was used to create the evaluation standards.

It is important to clarify that the six ‘criteria' referenced in the final rubric represent six thematic domains derived from the qualitative analysis, not a reduction of the 21 validated indicators. Each domain integrates multiple specific indicators that achieved expert consensus. Table 4 (provided below) details the distribution of the 21 specific indicators across the six domains.

**Table 4:** Distribution of the 21 Delphi-validated criteria across the six thematic domains.

Thematic domain	Criterion no.	Criterion summary (final wording after round 3)
1. Originality and creativity	1	Produces work with a wider and more diverse range of subjects, themes, and content than age-peers (merged with Criterion 19)
	3	Demonstrates flowing ideas and expansive imagination
	4	Demonstrates movement and form in artistic work
	16	Enjoys solving challenging and complex artistic tasks
	*Criterion 19*	*(Merged with Criterion 1)*
2. Technical proficiency and craftsmanship	6	Demonstrates precise use of color and contrast
	8	Exhibits high technical skill and craftsmanship
	9	Takes notes through drawing to document observations and ideas
	10	Demonstrates exceptional visual memory and observation skills through accurate recall and detailed depiction of visual information in artistic work *(revised in Round 3)*
3. Composition and design	2	Demonstrates compositional awareness and rich artistic vocabulary through intentional arrangement of visual elements and expressive use of artistic techniques *(revised in Round 3)*
	7	Assembles visual elements intelligently and consciously
	15	Shows interest in both figurative and abstract forms; demonstrates intuitive awareness of composition; moves fluidly between art mediums
	*Criterion 20*	*(Merged/revised into Criterion 2)*
4. Developmental appropriateness	11	Demonstrates effective integration of interaction, memory, and observation in artistic work
	18	Loves to understand and learn from artistic experiences and feedback
	21	Shows understanding of complex artistic phenomena and concepts beyond age level
5. Critical thinking and self-reflection	5	Leads teamwork and collaborates effectively in artistic projects
	13	Demonstrates self-criticism and a tendency to strive for perfection
	14	Shows strong and sustained interest in visual art
6. Commitment and motivation	12	Shows motivation and commitment to artistic tasks for extended periods
16	Enjoys solving challenging and complex artistic tasks
17	Commits to completing artistic tasks and overcomes difficulties in the artistic process

Regarding the criteria that required revision, Criteria 10, 19, and 20 had agreement rates below the 85% threshold in Round 2 (81.8%, 83.2%, and 82.1%, respectively). Based on expert feedback, Criterion 10 was revised to specify observable behaviors (‘Demonstrates exceptional visual memory and observation skills through accurate recall and detailed depiction of visual information in artistic work'), achieving 86.7% agreement in Round 3. Criterion 19 was identified as a duplicate of Criterion 1 and was merged with it, resulting in an enriched criterion that achieved 89.5% agreement. Criterion 20 was revised to operationalize the ambiguous term ‘rich artistic language' as ‘intentional arrangement of visual elements and expressive use of artistic techniques,' achieving 87.3% agreement. All revised criteria met the pre-established consensus threshold of 85% in Round 3 and were retained in the final scale. Table 4 presents the full mapping of the 21 Delphi-validated standards across the six thematic areas, illustrating how the specific indicators are organized to guide the development of the final rating scale.

As indicated in Table 4, Standard 19 was identified by experts as a duplicate of Standard 1 (“a broader and more diverse range of subjects”). Consequently, it was merged with Standard 1 during the third round; the combined standard now appears under Domain 1 (Originality and Creativity). In contrast, Standard 20 (“compositional awareness, rich artistic language”) was revised during the third round to address expert feedback regarding ambiguity. Its revised wording is now reflected in Standard 2 (“demonstrates compositional awareness and rich artistic language through the intentional arrangement of visual elements and the expressive use of artistic techniques”), which falls under Domain 3 (Composition and Design). Standard 10 (“Visual Memory and Excellent Observation”) was also revised during the third round to define observable behaviors; its revised wording is now reflected in Standard 10 under Domain 2 (Technical Competence and Craftsmanship). Additionally, Standard 16 appears in both Domain 1 (Originality and Creativity) and Domain 6 (Commitment and Motivation) because it addresses both creative engagement with a challenge (originality) and persistence in the face of difficulty (motivation). This cross-domain significance reflects the multidimensional nature of artistic giftedness.

### 6.2 Qualitative themes

Thematic analysis of written or recorded expert comments (from open-ended responses in the first round and justifications in the second and third rounds) identified six key themes, which corresponded precisely to the criteria's six sections. Themes have multiple sub-themes. Table 5 outlines these themes and provides typical expert quotations.

**Table 5:** Major themes, sub-themes and representative expert comments.

Theme	Sub-themes	Representative quote
1. Originality and creativity	Personal expression, imagination, creative problem-solving, risk-taking, breaking from tradition	“*The student shows technical competence, but his work doesn't have a genuine voice; it looks as if it was dictated to him.”*
2. Technical proficiency and craftsmanship	Media control, detail/precision, media variety, rapid learning, implementation quality	“*He handles the brush with professionalism.” “His work looks professional for his age.”*
3. Composition and design	Balance, use of space, line/form, texture, compositional innovation	“*The work is exceptionally well-balanced.” “It uses negative space expertly.”*
4. Developmental appropriateness	Peer progression, gifted progression, developmental acceleration, artistic maturity, age gap	“*His work is more advanced than that of his classmates.” “He draws with perspective usually seen at an older age.”*
5. Critical thinking and self-reflection	Describing work, justifying choices, analyzing strengths/weaknesses, connecting elements to meaning, critiquing others	“*He knows what he did well and what he needs to improve.” “He connects negative space with mood.”*
6. Commitment and motivation	Perseverance, focus/engagement, intrinsic motivation, self-confidence, overcoming challenges	“*He doesn't give up despite the difficulty.” “His motivation is a love of art, not because someone asked him to.”*

Table 5′s primary themes and sub-themes were utilized to develop descriptors of the level of performance (unsatisfactory, emerging, competent, perfect) for each of the six final criteria.

### 6.3 The final rating scale: gifted rating scale—artistic talent (GRS-AT)

The rating scale was developed by combining quantitative consensus (≥85% agreement on all 21 criteria) with qualitative themes (major and minor). The 21 identified criteria were organized into six thematic domains through a multi-stage process. In the first round, a thematic analysis of expert responses identified 21 sub-themes. In the second round, experts evaluated each sub-theme as a specific criterion. In the third round, expert feedback identified overlaps and redundancies, leading to the merging of duplicate criteria (19 with 1, and 21 with 3) and the revision of ambiguous criteria (10 and 20). The remaining criteria were then grouped by thematic domain, and integrated performance descriptors were developed for each domain based on expert feedback and theoretical frameworks.

The rating scale consists of six domains, each evaluated on a four-point scale (0 = Insufficient, 1 = Developing, 2 = Proficient, 3 = Exemplary). Total scores range from 0 to 18. The evaluation levels are:

- 0–6 = Insufficient (little to no evidence of artistic talent).
- 7–11 = Developing (some indicators, potential for development).
- 12–15 = Proficient (clear indicators and potential talent).
- 16–18 = Exemplary (exceptional indicators across all categories, exceptional talent).

Teachers must evaluate students using the six general rating scale levels (domains), rather than the 21 individual sub-criteria. The 21 sub-criteria served as research-validated indicators that informed the development of the rating scale and can be used as behavioral anchors for teacher observation and coaching; however, they are not intended for individual assessment in classroom practice. Table 6 presents the abridged rating scale; the full rating scale is available in Appendix 1.

**Table 6:** Summarized assessment criteria and performance levels in the GRS-AT system.

Criterion	Unsatisfactory (0)	Emerging (1)	Proficient (2)	Exemplary (3)
1. Originality and creativity	No individual expression, dependent on models	Constrained personal expression, few creative ideas	Modest personal expression, ingenuity, innovative problem-solving	Strong original expression, vivid imagination, risk-taking, breaks from tradition
2. Technical proficiency and craftsmanship	No media control, lack of attention to detail, untidy	Little media control, insufficient detail, inconsistent execution	Adequate media control, tidy execution	Advanced media management across several media, exceptional detail, professional-looking
3. Composition and design	Disorderly, lack of balance, uncertain lines	Conventional application, developing awareness of balance	Traditional yet adept application, effective use of space	Original and distinctive amalgamation, proficient use of balance, negative space, inventive compositions
4. Developmental appropriateness	Below age expectations	Meets age expectations, equivalent to average peers	Above average, outperforms same-age peers	Remarkable, outperforms gifted peers, abilities typical of older students
5. Critical thinking and self-reflection	Cannot describe or justify choices, no self-evaluation	Briefly describes, little reasoning, superficial self-assessment	Explains artwork, provides logical justifications, identifies strengths/weaknesses	Coherent descriptions, links formal elements to meaning, outstanding self-understanding
6. Commitment and motivation	Gives up quickly, works superficially, avoids challenges	Completes tasks but little persistence, easily sidetracked	Persists with adversity, maintains focus, launches own tasks	Exceptional tenacity, profound absorption/flow, intrinsically motivated, seeks demanding subjects

As shown in Table 6. The Delphi approach produced a rubric-based assessment with 21 criteria across six areas. After three rounds, all criteria met the specified 85% agreement criterion. The qualitative study confirmed six important areas that corresponded to the assessment criterion domains. The final GRS-AT contains an organized and linked criterion rubric for identifying artistically talented kids, with four performance levels per subject.

## 7 Discussion

The current study was out to identify artistically gifted kids in Saudi Arabia's middle and high schools. The Artistic Gifted Rating Scale-AT (GRS-AT) is an evidence-based, criterion-referenced rubric. Using a modified Delphi method, 120 experts met in three rounds to reach a strong agreement on 21 criteria across six domains: originality and creativity, technical proficiency and craftsmanship, composition and design, developmental relevance, critical thinking and self-reflection, and commitment and motivation. All criteria achieved the acceptable level of agreement (85%). The final scale provides a structured, performance-based metric that meets a critical need in the evaluation of Saudi arts education.

Criterion 18 received the most responses: “Artistically gifted children enjoy solving challenging artistic tasks” (62.5% strongly agreed, 90% generally agreed). This data shows that professionals see a love of challenge and commitment to the task as the greatest distinguishing features of artistic creativity, surpassing technical proficiency and originality. This conclusion suggests that these experts believe the love of challenge and task devotion to be among the most prominent characteristics of artistic genius, outweighing other features such as technical proficiency and inventiveness. This viewpoint is consistent with Renzulli's ‘Three-Ring Conception of Giftedness,' which lists task commitment as one of the three core components of giftedness, along with above-average ability and creativity (Renzulli and Reis, 2014). In contrast, the experts believe that the pursuit of challenges in the artistic domain reflects not only determination and perseverance, but also a genuine inclination toward complexity; ‘these gifted individuals deliberately choose complex subjects' and ‘enjoy engaging in artistic endeavors that involve challenge'. Renzulli's theory supports this conclusion, and thus it can be generalized to include the visual arts, confirming the need for artistic giftedness assessment methods to include criteria that highlight the student's enthusiasm for challenging tasks, rather than simply measuring their current level of ability (Renzulli and Reis, 2014).

### 7.1 Creative expression valued over technical skill alone

Technical proficiency emerged as a major focus (92.5% agreement on the eighth criterion); however, qualitative analysis revealed a slightly lower importance, as experts considered authentic creative expression to be a more accurate indicator of talent, while viewing technical skill as a necessary but insufficient prerequisite for assessing a student's potential. This contrast was reaffirmed by one expert, who described it as: ‘The pupil exhibits technical proficiency; however, their work lacks an authentic voice; it looks to be the product of dictation and rote learning rather than a self-generated creative process.' This study confirms Gardner's emphasis on the aspect of 'sensing', which he characterized as the ability to go from the literal to the symbolic levels, or to see 'beyond the surface (1989). It also supports Clark and Zimmerman's (2004) statement that realistic drawing ability is one of numerous indications of creative potential. In practice, checklists that favor drawing correctness or technical prowess may overlook highly creative students whose works, while less technically polished, stand out for their distinctive substance and concept.

### 7.2 The metacognitive dimension of artistic talent

The topic of critical thinking and self-reflection was very prominent in our findings, with Criterion 13 (“self-critical, perfectionist”) scoring 97.5% agreement, the second highest. Experts stressed the importance of students' capacity to articulate their work, justify their creative decisions, identify their own strengths and limitations, and relate formal characteristics to meaning—for example, “connects negative space to mood.” This coincides with the transition to constructivist cognitive theory, which supports engaging talented kids in self-evaluation and metacognitive thinking (Aboud, 2023a, 2025; Brajčić et al., 2018). This is clearly incorporated into our scale via the Critical Thinking and Self-Reflection criterion, making it one of the few geographically specific assessment instruments that assesses a student's capacity to view their own artwork as a process rather than a product.

### 7.3 Developmental acceleration as a key indicator

Several studies have highlighted developmental acceleration as a hallmark of giftedness (Plucker et al., 2021; Kettler and Taliaferro, 2022). This is further confirmed by our study's subthemes, which include peer-to-peer progression (“his work is more advanced than his classmates”), inter-given development (“even among gifted students”), and age gap (“it looks like the work of a true artist”). This is consistent with Jarosewich et al.'s (2002) discovery that gifted children achieve developmental milestones earlier than their classmates. In the visual arts, this manifests as drawing in perspective at a younger age, understanding depth earlier, and producing work comparable to that of older students or even adults. Including developmental relevance in the scale (criterion 4) provides educators with an orderly means to compare student work to expected age-appropriate standards—an important but usually forgotten feature of the assessment process.

### 7.4 Affective domain: commitment and motivation

The non-cognitive traits of artistically gifted children, in general, were addressed in the sixth section, entitled ‘Commitment and Motivation'. These students, as described by the expert participants in the study, possess intrinsic motivation and spontaneity—they ‘love art for its own sake, not because someone asked them to'—and have a remarkable capacity for concentration ‘that reaches the point of complete immersion and obliviousness to their surroundings,' as well as perseverance in the face of obstacles. Many of these characteristics align with the scientific literature on giftedness, which emphasizes 'a strong passion for learning‘ and 'an intense desire for mastery‘ as important criteria (Clark and Zimmerman, 2004). Al-Ghamdi's (2022) study supports this view, indicating that most Saudi art students possess a genuine passion for the arts, even in the face of weak institutional support for their artistic skills. Accordingly, our scale's focus on innate motivation and perseverance may help identify gifted young people who continue to practice art despite the absence of institutional support, thereby revealing traits that would otherwise have remained hidden. This finding is of critical importance, as it highlights a fundamental gap between students' self-motivation and the institutional resources available in the field of art education in the Kingdom of Saudi Arabia. In this context, Al-Ghamdi's (2022) study revealed that despite limited opportunities and support directed toward the arts, the vast majority of Saudi art students continue to engage in their artistic creativity; they provide their own art supplies, launch their own projects, and dedicate their personal time to artistic practice.

### 7.5 Comparison with existing tools

The GRS AT scale is designed to address the issue of teachers being unprepared to support artistically gifted kids by providing art teachers with dependable methodological criteria. It is intended to allow teachers to discover pupils who may have hidden artistic abilities, a concern raised by various researchers, including Abdulhafeez (2021) and Al-Ghamdi (2022). The GRS AT's emphasis on process, metacognition, and intrinsic motivation is consistent with larger educational objectives of producing critical and creative thinkers for a knowledge-based economy.

The GRS AT scale differs from other methods for identifying artistically gifted people in various respects. Unlike Clark's Drawing Ability Test (Clark and Wilson, 1991), which requires individuals to complete a standardized drawing task focused primarily on realistic representation and evaluates their artwork, our scale is a criterion-referenced performance assessment that evaluates multiple domains (creativity, composition, critical thinking, and motivation) in addition to Clark's drawing accuracy requirement. Our scale's larger definition of creative skill is more consistent with current multi-criteria theories of giftedness. Furthermore, unlike the Gifted Rating Scale—Fourth Edition (GES 4, McCarney and House, 2018), which has only one subscale for “Performing and Visual Arts,” the GRS AT has many criteria that cover all aspects of the visual arts. The scale we use also includes specific behavioral characteristics for all four performance levels, making it more relevant for classroom teachers. Finally, unlike the Gifted Rating Scales (GRS), which screen giftedness across six domains, including artistic potential, by stating traits such as “has a good imagination” (Jarosewich et al., 2002; Aboud, 2023a), the GRS AT follows alternative assessment concepts (Wiggins, 1993). It necessitates systematic observation of both the process and the outcome, integrates self-reflection, and provides qualitative descriptions to guide the teacher's evaluation, rather than a checklist.

### 7.6 The GRS AT uses rubrics to evaluate projects and portfolios

The GRS AT scale is designed to address the issue of teachers being unprepared to support artistically gifted kids by providing art teachers with dependable methodological criteria. It is intended to allow teachers to discover pupils who may have hidden artistic abilities, a concern raised by various researchers, including Abdulhafeez (2021) and Al-Ghamdi (2022). The GRS AT's emphasis on process, metacognition, and intrinsic motivation is consistent with larger educational objectives of producing critical and creative thinkers for a knowledge-based economy.

### 7.7 Cultural considerations and limitations to generalizability

Although the GRS-AT scale was designed by Saudi professionals and is intended for use in Saudi schools, certain cultural concerns deserve specific recognition, and statements about generalizability must be adjusted accordingly. First, the Delphi panel's understanding of artistic giftedness, as expressed in the GRS-AT criteria, was influenced by the cultural and educational milieu. The criteria value individual expression, uniqueness, risk-taking, and vocal articulation of artistic choices, which match with Western ideas of creativity. However, Saudi creative traditions have historically emphasized specific values, such as geometric precision, calligraphy expertise, pattern-based composition, and decoration, which modern standards may not fully portray. The extent to which the GRS-AT scale criteria accurately depict quality in traditional Saudi and Islamic art forms has not been thoroughly investigated, and this is an open subject for future research. Secondly, societal principles of collectivism as opposed to individualism may affect the expression and assessment of artistic talent. In the collectivist society of Saudi Arabia, characteristics like “risk-taking” and “straying from tradition”—recognized as markers of innovation in our evaluation framework—might be manifested more discreetly than in individualistic Western cultures. In a similar vein, “self-criticism” and “critical thinking" could be influenced by cultural standards that prioritize deference to educators and authoritative figures, which may impact the manner in which students express their artistic decisions and partake in self-examination. The present study did not directly examine these cultural subtleties, which may affect teachers' interpretations and applications of the grading scale across various Saudi subcultures or areas. Third, the GRS-AT scale's emphasis on the verbal articulation of artistic choices (Criterion 5: Critical Thinking and Self-Reflection) may favor students who are verbally fluent and culturally comfortable with self-expression, potentially disadvantageous to students from cultural backgrounds that value humility and respect in educational settings. This represents a potential bias that should be investigated in future research.

Fourth, when generalizing the GRS-AT scale results beyond Saudi Arabia, proceed with caution. Although the Delphi panel included professionals from a variety of professional backgrounds, including academics, professional artists, and art critics, all were based in Saudi Arabia, and their decisions reflected the country's unique artistic and educational milieu. The criteria's relevance to other Gulf Cooperation Council (GCC) states, Arab nations, or non-Arab contexts has yet to be determined; this will require comprehensive cross-cultural validation studies. While the scale's conceptual framework and validation procedure could be used as a template for similar initiatives elsewhere, the specific criteria and performance descriptors should be tailored to local creative traditions, cultural values, and educational interests.

## 8 Limitations

Although the scale for assessing creative talent in gifted students was designed through expert consensus, it has yet to be field tested. While the scale has exhibited content validity, its predictive validity—the relationship between scale scores and future creative accomplishment among artistically talented students—has yet to be proved. As a result, future research should focus on the scale's classroom application and its capacity to distinguish between two groups of children who have been pre-identified as artistically gifted vs. average. Given the urgent need for a comprehensive assessment, the length of the scale—which includes 21 criteria distributed across six domains—may pose a practical challenge for time-constrained classroom teachers; however, training and practice can play a significant role in progressing through the assessment more quickly and efficiently.

Moreover, the study did not fully address the issue of cultural biases. Although the expert panel included Saudi teachers, art critics, and professional artists, their evaluation is influenced by Western artistic traditions—such as realism, perspective, and individual expression—rather than Saudi artistic traditions, such as Islamic geometric ornaments, calligraphy, and pattern-based composition. This potential cultural bias is a significant limitation, as it may result in the under identification of students whose artistic strengths lie in traditional or non-Western art forms. Future studies concerned with cross-cultural adaptation should systematically examine the applicability of these criteria to the types of Eastern and Islamic arts prevalent in Saudi Arabia and explore whether additional criteria are needed to capture excellence in these traditions.

Furthermore, the study did not explicitly address the role of cultural values such as collectivism, modesty, and respect for authority, which may influence how artistic giftedness manifests and how teachers assess it. The emphasis on verbal self-expression and self-criticism in the rubric may favor students from certain cultural backgrounds while disadvantaging others. Future research should investigate these cultural dynamics and explore whether the rubric's performance descriptors require cultural adaptation for different Saudi subpopulations. Finally, the study employed the Delphi methodology, which is characterized by its reliance on a large and diverse panel of experts (including faculty members from different countries); however, the extent to which it can be applied in other Gulf or Arab contexts requires further testing and validation. The criteria should be considered context-specific rather than universally applicable.

## 9 Recommendations for future research and practice

To address the cultural limitations identified above, we propose the following recommendations:

First, we recommend that future research develop culturally adapted versions of the GRS-AT that incorporate performance descriptors specific to traditional Saudi and Islamic art forms—including geometric patterns, calligraphy, and ornamentation—while maintaining the core six-domain structure validated through the Delphi process. This adaptation would involve: (a) replacing Western-oriented descriptors such as “realistic depiction,” “perspective,” and “individual expression” with culturally relevant alternatives such as “geometric precision,” “proportional accuracy,” and “mastery of calligraphic line”; (b) developing exemplars of student work at each performance level for each traditional art form; and (c) conducting validation studies with specialists in traditional Islamic arts to ensure cultural authenticity and appropriateness.

Second, teachers applying the rubric in Saudi classrooms should receive targeted professional development to interpret each domain in light of traditional artistic values. Training should emphasize that “innovation” within Islamic art traditions may take the form of subtle variation within established patterns rather than radical departure from tradition, and that “technical proficiency” may manifest as geometric precision, proportional accuracy, and calligraphic mastery alongside realistic representation. Such cultural sensitivity is essential to ensure that the GRS-AT supports the identification and development of all artistically gifted students, regardless of whether they work within Western or traditional Islamic art forms.

Third, future research should investigate how cultural values such as collectivism, modesty, and respect for authority influence the manifestation of artistic giftedness in Saudi students and how these values should inform the interpretation of the GRS-AT performance descriptors. This may involve qualitative studies with teachers and students, cross-cultural comparisons, and the development of culturally responsive assessment practices.

Fourth, we recommend that cross-cultural validation studies be conducted to examine the applicability of the GRS-AT in other Gulf Cooperation Council countries and Arab contexts. Such studies should assess whether the criteria require further adaptation to reflect local artistic traditions, cultural values, and educational priorities.

Finally, we encourage researchers and practitioners to view the GRS-AT as a flexible framework that can be adapted to diverse cultural contexts, rather than a fixed instrument. The core six-domain structure—originality and creativity, technical proficiency and craftsmanship, composition and design, developmental appropriateness, critical thinking and self-reflection, and commitment and motivation—provides a comprehensive foundation that can be meaningfully interpreted across different artistic traditions with appropriate cultural adaptation.

## 10 Conclusion

In this study, we created a consensus-based, criterion-referenced scale (GRS-AT) to identify artistically gifted adolescents at intermediate and secondary school levels in Saudi Arabia. Using the Delphi technique, we constructed 21 criteria divided into six categories, all of which achieved an 85% consensus. The findings show that artistic giftedness is complex, incorporating creative, technical, compositional, developmental, and affective dimensions, as well as linkages to metacognition, and that reducing it to the ability to draw realistically is unjust. By introducing a systematic talent identification tool specifically designed for the visual arts, the GRS-AT scale addresses a critical need within art assessment systems in Saudi Arabia. Its added value—distinguishing it from traditional IQ tests and checklists—lies in its use of alternative, performance-based assessment principles that simultaneously emphasize the creative process and self-reflection. Within the context of art education in Saudi Arabia, the scale offers an effective mechanism for achieving educational goals related to creativity and cultural development, while also helping to identify and nurture previously undiscovered gifted students. However, it is important to emphasize that the GRS-AT requires further empirical validation through field testing before it can be considered a fully validated instrument for widespread implementation.

## Data Availability

The data analyzed in this study is subject to the following licenses/restrictions: All data is displayed in the manuscript. Requests to access these datasets should be directed to yozaki@kfu.edu.sa.
