# Supplementary material for: Beyond the canvas: a Delphi-driven rubric for unlocking artistic giftedness
Source: Front Psychol. 2026 Sep 23;17:1961771. doi: 10.3389/fpsyg.2026.1961771 (PMC13642194; doi:10.3389/fpsyg.2026.1961771)
Supplement: Supplementary file 1 [file Table_1.docx]

**Appendix 1**

Proposed Rubric: Assessment of Artistically Gifted Students in Visual Arts (Developed Through Expert Consensus)

| Criteria | Unsatisfactory (0 points) | Emerging (1 point) | Proficient (2 points) | Exemplary (3 points) | Rating |
| --- | --- | --- | --- | --- | --- |
| 1. Originality and creativity: personal expression, imagination, creative problem-solving, risk-taking, avoiding imitation | The artwork has no signs of individual expression and is wholly dependent on the emulation of models or instructor examples. | The artwork exhibits constrained personal expression; it has a few creative concepts but is predominantly conventional. | The artwork showcases modest personal expression, ingenuity, and innovative problem solving. | The artwork exemplifies strong original creative expression, vivid imagination, innovative problem-solving, risk-taking, and departures from traditional methodologies |  |
| 2. Technical Proficiency and Craftsmanship: Media Control, Detail/Precision, Media Variety, Rapid Learning, Implementation Quality | Artistic abilities are lacking; there is no control over media; there is a lack of attention to detail; and the execution is untidy. | Artistic abilities are lacking, with little media control, insufficient attention to detail, and inconsistent execution. | Artistic abilities are average; there is adequate media control and attention to detail; tidy execution. | Artistic abilities are advanced and prominent; great media management across several media; exceptional attention to detail; precise, clean, professional-looking execution for the age. |  |
| 3. Composition and Design: Balance, spatial utilization, line/form, texture, compositional originality. | Disorderly use of artistic elements; lack of balance or spatial coherence; lines exhibit uncertainty. | Conventional application of artistic elements; developing awareness of balance and spatial relationships; lines have a utilitarian purpose. | Traditional yet adept application of artistic component; effective equilibrium and utilization of negative space; assured line work. | Original and distinctive amalgamation of creative components and design tenets; proficient application of balance, negative space, and texture; assertive, expressive lines; inventive compositions. |  |
| 4. Developmental Suitability: Peer advancement, talented advancement, developmental acceleration, creative sophistication, age disparity | The artwork falls below age-appropriate expectations. | The artwork meets age-appropriate requirements and is equivalent to average peers of the same age. | The artwork is above average; it outperforms peers of the same age group and has some advanced skills for its age. | The artwork is remarkable; it outperforms gifted peers of the same age; it exhibits abilities typical of older pupils; and it reveals artistic maturity beyond chronological age. |  |
| 5. Critical Thinking and Self-Reflection: Describing work, defending choices, examining strengths and flaws, connecting elements to meaning. | Cannot define the artwork or explain the reasoning behind artistic decisions; no sign of self-evaluation. | Can briefly describe the artwork; recognizes aspects employed; provides little reasoning for choices; performs superficial self-assessment. | Able to explain the artwork, provide logical justifications for artistic decisions, and point out some of its advantages and disadvantages. | Can express coherent descriptions, reasons, and self-criticism; links formal elements to meaning/mood; exhibits outstanding understanding of one's own artistic decisions. |  |
| 6. Commitment and Motivation: Perseverance, focus/engagement, intrinsic motivation, self-confidence, and challenge-seeking. | Gives up quickly; works superficially; produces just when necessary; avoids problems; conceals work. | Completes things but exhibits little persistence; is easily sidetracked; requires external suggestions; and is hesitant to share work. | Persists in the face of adversity; maintains focus; launches some tasks on their own; is prepared to share labor; and embraces moderate obstacles. | Exceptional tenacity with difficult projects; profound absorption/flow; innately motivated (creates for the love of art); actively seeks out demanding subjects; and confident in showing work. |  |

***Note: Scoring Guide for the GRS-AT***

*Each of the six criteria is rated on a scale of 0 to 3 points, with 0 = Unsatisfactory, 1 = Emerging, 2 = Proficient, and 3 = Exemplary. Total scores range from 0 to 18 points.*

***Score Interpretation:***

- *0-6 points: Unsatisfactory – Little or no evidence of artistic talent; may need further observation or support*
- *7-11 points: Emerging – Shows some signs of artistic talent; potential may be developing*
- *12-15 points: Proficient – Shows clear signs of artistic talent; likely talented*
- *16-18 points: Outstanding – Shows exceptional signs across all criteria; profoundly talented*
